# Radioprotective effect of Date syrup on radiation- induced damage in Rats

**DOI:** 10.1038/s41598-018-25586-3

**Published:** 2018-05-09

**Authors:** Shimaa M. Abou-Zeid, Badr E. EL-bialy, Nermeen B. EL-borai, Huda O. AbuBakr, Abdel Monsef A. Elhadary

**Affiliations:** 1 Department of Forensic Medicine and Toxicology, Faculty of Veterinary Medicine, University of Sadat City, Sadat city, Egypt; 2 Department of Biochemistry and Chemistry of Nutrition, Faculty of Veterinary Medicine, Cairo University, Giza, 12211 Egypt; 3 Atomic Energy Authority, Cairo, Egypt

## Abstract

Ionizing radiation has cytotoxic and genotoxic effects caused mainly by the oxidative damage induced by free radical release. The need for radioprotectives is increasing to protect normal tissues during radiotherapy. In the present study, we investigated the radioprotective effect of Date syrup in rats subjected to whole body radiation at 6 Gy through biochemical, molecular and histopathological analysis. Significant elevations were recorded in the activities of serum ALT, AST, ALP and LDH and in the levels of all lipid profiles parameters, while the level of HDL-C was reduced. The concentration of liver MDA was elevated with depletion of hepatic glutathione (GSH) and catalase. DNA damage was evidenced by increased DNA strand breakage and DNA-protein crosslinks. Significant elevations were observed in the expression of liver TNF-α and serum activity of matrix metalloproteinase (MMP-9). Pretreatment of rats with Date syrup ameliorated the tissue damage induced by radiation as evidenced by the improvement of liver function, antioxidant status and reduction of DNA damage. Besides, liver TNF-α expression and serum MMP-9 activity were reduced. In conclusion, Date syrup could alleviate the toxic effects of ionizing radiation and thus is useful as a radioprotective in radiotherapy regimen.

## Introduction

Ionizing radiation results in an array of biological consequences, including inflammation, carcinogenesis, and death. Exposure of humans and animals to radiation occurs through radiotherapy, experimentation, work in nuclear stations, nuclear battlefields and nuclear accidents^1^.

Ionizing radiation induces oxidative stress as a result of increased production of free radicals which attack various components in the cell leading to biochemical changes and macromolecule modifications such as lipid peroxidation, protein oxidation, and DNA strand breaks^2,3^.

Moreover, reactive oxygen species (ROS) have a negative effect on the cellular antioxidant defense mechanisms by reducing the level of reduced glutathione (GSH) and the activity of antioxidant enzymes, especially catalase (CAT), superoxide dismutase (SOD) and glutathione peroxidase (GPx)^4,5^. In addition, some harmful oxidative molecules such as malondialdehyde (MDA), nitrite/nitrate (N/N), advanced oxidation protein products (AOPP) and ischemia-modified albumin (IMA) are produced during ionizing radiation reactions^6,7^.

Likewise, matrix metalloproteinases (MMPs); family of calcium-dependent, zinc-containing endopeptidases that includes stromelysins (1,3) collagenases (1,3,8), gelatinases (2,9) and membrane type1 MMP^8^ are induced by proinflammatory cytokines such as tumor necrotic factor alpha (TNF-α), interleukin 1β (IL-1β) and several growth factors during oxidative stress cascade^9,10^. Meanwhile, the balance between MMPs and their regulatory tissue inhibitors of metalloproteinases (TIMPs) are shifted toward the transcription of MMPs causing degradation of extracellular matrix (ECM) leading to diverse pathological conditions^11^.

Although radiotherapy is beneficial in controlling tumors in human and animals, it induces damages to the bone marrow, liver and other tissues. These limit the therapeutic dose of radiation that is essential to control tumors^1^. Therefore, the use of radioprotectives is of great importance to protect normal tissues from the expected damage induced by radiation. Because free radicals are the main cause of irradiation-induced cellular damage, antioxidants and free radical scavengers are expected to act as radioprotectors. This causes biologists and radiation oncologists seek continuously for radioprotectors.

There is a growing interest in the use of naturally occurring antioxidants as radioprotectors. Ajwa date is the fruits of the female tree Date palm (Phoenix dactylifera L.). It is very commonly consumed in Egypt, Arab countries and many other parts of the Middle East. This fruit contains valuable amounts of carbohydrates, salts, minerals, dietary fibers, vitamins, fatty acids, amino acids, and protein giving the fruit significant nutritional value^12^. This fruit has many useful properties including antioxidant, anticlastogenic, antidiabetic, hypolipidemic, anticarcinogenic, antibacterial and antifungal activities. In addition, the ethanolic and watery extracts of date fruit were useful in reducing the severity of nephrotoxicity, neurotoxicity and gastric ulceration via antioxidant mechanisms^13,14^. Date syrup (debis) is prepared by keeping Date fruit pieces in water overnight, then boiling the mixture for 2 hours followed by filtration^15^. Another method includes mixing the fruits with ethanol 80% and extraction is carried out for 30 min at 80 °C followed by filtration^16^.

The hepatoprotective effect of Date fruit extract was previously demonstrated against the oxidative damage induced by many toxicants including CCl_4_^17^, thioacetamide^18^, trichloroacetic acid^19^ and dimethoate^20^. However, to the best of our knowledge, the antioxidant activity of Date syrup was only studied *in vitro*^15^ and no available studies explored the protective and antioxidant activity of date syrup against poisons in an experimental model.

Therefore, the present investigation was performed to investigate the possible protective effect of Date syrup against total body radiation induced harmful effects in rats.

## Materials and Methods

### Chemicals

Date syrup was purchased from Al Tahhan Company, Alwadi Elgadid, Egypt. Kit for serum lactate dehydrogenase was purchased from Salucea (Netherlands). Kits for aminotransferases, alkaline phosphatase, lipid profile and oxidative stress evaluation were purchased from Biodiagnostic (Egypt).

### Animals and Treatment

This study was carried out in strict accordance with the recommendations in the Guide for the Care and Use of Laboratory Animals approved by the Committee on the Ethics of Animal Experiments of University of Sadat City, Egypt.

Male Wistar albino rats (200 ± 10 g) were purchased from the Animal Care Unit of Vacsera Pharmaceutical Company, Agouza, Egypt. Animals were housed in plastic cages with stainless-steel grid tops and kept at a room with standard conditions (a 12-h light/dark cycle; temperature maintained 23 ± 2 °C). The animals received standard diet and water *ad libitum*.

Animals were divided into 4 groups, 15 rats each. Group 1 (Control); received 1 ml 0.9% saline solution orally for 4 weeks and served as control; Group 2 (Irradiated); was exposed to radiation at a dose level of 6 GY^21^ and sacrificed after 48 hours. Group 3 (Date syrup); received daily Date syrup by stomach intubation at a dose of 4 ml/kg body weight^20^ for 4 weeks; Group 4 (Irradiated + Date syrup); was pretreated as group 3 for 4 weeks and then irradiated as group 2 and sacrificed 48 hours after irradiation.

Animals of each group were starved for 12 hours, anesthetized by a combination of Ketamine 50 mg/kg BWt, Xylazine 5 mg/kg BWt and Thiopental sodium 50 mg/kg BWt. Blood samples were collected from the medial canthus of eye and left to clot in a clear dry centrifuge tubes, then centrifuged at 3500 rpm for 15 minutes for serum preparation and stored at −20 °C for further biochemical studies. Then rats were scarified and liver samples were collected and divided into two portions; first one stored at −80 °C for assessment of oxidant/antioxidant biomarkers, pro-inflammatory mediator and gene expression. Other portion of liver samples were fixed in 10% neutral formalin and prepared for histopathological examination.

### Irradiation procedure

Whole-body gamma-irradiation was performed at the Atomic Energy Authority, Cairo, Egypt, using a Gamma Cell-40 Carloirradiator, cesium137 source. Animals were irradiated at an acute single dose level of 6 Gy delivered at a dose rate 0.713 rad/sec.

### Serum biochemical analysis

Activities of serum aminotransferases^22^, lactate dehydrogenase^23^ and alkaline phosphatase^24^ were determined following manufacturer’s instructions. The concentrations of total cholesterol^25^, triglycerides^26^ and high-density lipoprotein-cholesterol “HDL-C”^27^ were determined using commercially available kits following the manufacturer’s instructions. Serum levels of low-density lipoprotein-cholesterol (LDL-C) and very low-density lipoprotein-cholesterol (VLDL-C) were calculated according to the equations of Lee and Nieman^28^ as follows:

$${\rm{LDL}}\,({\rm{mg}}/{\rm{dl}})={\rm{TC}}-{\rm{HDL}}-{\rm{TG}}/{\rm{5}}$$

$${\rm{VLDL}}\,({\rm{mg}}/{\rm{dl}})={\rm{TG}}/{\rm{5}}$$

### Assessment of oxidant/antioxidant biomarkers

#### Lipid peroxidation

Malondialdehyde (MDA) concentration was used as the index of lipid peroxidation as described by Ohkawa *et al*.^29^. It was determined by measuring the thiobarbituric acid reactive species. The absorbance of the resultant pink product was measured at 532 nm.

#### DNA damage

Comet assay: Parts of liver from the four groups were washed in cold buffer (NaCl; 75 mmol/I, EDTA-2Na; 24 mmol/I, PH 7.5) and minced with a pair of scissors and homogenized using a potter-type homogenizer. The cell suspensions were centrifuged at 4 °C, 700 × g for 10 min. The supernatants were removed, and the cells were resuspended in cold buffer. The slides were prepared according to Singh *et al*.^30^ and examined at 100x magnification under a fluorescence microscope using a FITC filter. The level of DNA damage was determined in each slide by evaluating tail length “µm”, % DNA in tail and tail moment.

Evaluation of DNA-protein crosslinks (DPCs) percentages: DNA-protein crosslinks were determined according to the procedures described by Zhitkovich and Costa^31^. Addition of KCl to SDS resulted in the formation of an insoluble precipitate (K-SDS) that easily recovered by centrifugation. So, DNA that was crosslinked with proteins was precipitated leaving free DNA in the supernatant. Pretreatment of lysed cells with proteinase K clearly distinguishes DNA with and without DPCs. the free and crosslinked DNA in the pellet after treatment with PK were used for quantification of DPCs by the Diphenylamine. The developed blue color was colormetrically quantified spectrophotometrically at 578nm. The Percentage of DPCs in each sample was expressed by the formula: DPCs% = (O.D Supernatant/O.D Supernatant + O.D Pellet) × 100. (O.D. optical density).

#### Glutathione reduced (GSH

Assessment of glutathione reduced (GSH) depends on the reduction of 5,50-dithiobis 2-nitrobenzoic acid with glutathione producing a yellow color whose absorbance is measured at 412 nm according to Beutler *et al*.^32^.

#### Catalase activity (CAT)

Catalase reacted with a known quantity of H_2_O_2_ and the reaction is stopped after 1 min with catalase inhibitor. In the presence of peroxidase, the remaining H_2_O_2_ reacts with 3,5-Dichloro-2-hydroxybenzene sulfonic acid and 4-aminophenazone to form a chromophore with a colour intensity inversely proportional to the amount of catalase in the sample. The absorbance was measured at 510 nm as described by Aebi^33^.

### Assessment of matrix metalloproteinase-9 activity (MMP-9)

The activity of MMP-9 was detected in gelatin zymography by a method described by Hawkes *et al*.^34^. Briefly, serum samples were separated by SDS/PAGE on 7.5% (w/v) gels, containing 1 mg/ml gelatin under non-reducing conditions. Then, it was washed twice for 15 min each in 2.5% (v/v) Triton X-100 and incubated in development buffer (0.05 M Tris/HCl, pH 8.8, 5 mM CaCl_2_, 0.02% NaN_3_) for 15 min to overnight incubation. Gels were stained with 0.1% Coomassie Brilliant Blue R250 in methanol:acetic acid:water (4.5:1:4.5, v/v/v). The zymograms gels were scanned in true colour and then analyzed using commercially available software (myImageAnalysis Software; ThermoscientificTM) after conserving to grey scale.

### Quantitative real- time PCR evaluation for TNF-α gene expression

#### Total RNA extraction and cDNA synthesis

Total RNA was extracted from liver samples using the Purelink RNA extraction kit (Invitrogen) according to the manufacturer’s protocol. The concentration and purity of the total RNA samples were obtained by using a Nanodrop ND-1000 spectrophotometer. cDNA was synthesized using the Maxima first-strand kit (Invitrogen) according to the manufacturer’s recommendations. The synthesized cDNA samples were stored at −20 °C until further use.

#### Real-time qPCR and gene expression analysis

Real-time PCR (qPCR) was carried out using the reaction mixture of 1 μl cDNA, 0.5 mM of each primer (TNF-α and GAPDH as an internal control), iQ SYBR Green Premix (Bio–Rad 170–880, U.S.A.) in a total volume of 20 μl. PCR amplification and analysis were achieved using Bio–Rad iCycler thermal cycler and the MyiQ realtime PCR detection system. All templates were amplified using the following Lightcycler protocol. The primers for TNF-α were based on the sequence published in gene bank NM_012675.3. One for Rattus norvegicus; forward primer: ACACACGAGACGCTGAAGTA and the reverse one: GGAACAGTCTGGGAAGCTCT. The fast start polymerase was activated and cDNA denatured by pre-incubation for 15 min at 95 °C, the template was amplified for 40 cycles of denaturation programmed for 45 s at 95 °C, annealing of primers at 62 °C programmed for 45 s and extension at 72 °C programmed for 10 min. Fluorescent data were acquired during each extension phase. Each assay includes triplicate samples for each tested cDNAs and no-template negative control. Data analysis of the relative gene expression ratio for measuring the change in the expression level of a gene was calculated by the cycle threshold (ΔΔCT) method according to the manufacturer’s recommendations^35^. The data were normalized using GAPDH as the reference housekeeping gene.

### Histopathological examination

Liver samples were fixed in 10% neutral formalin and prepared for histopathological examination according to Banchroft *et al*.^36^. Sections were microscopically scored and derived semi-quantitatively as following: −, None; +, slight <20%; ++, moderate <50%; +++, severe > 50% of examined sections.

### Statistical analysis

The obtained values are given as means ± S.E of the mean. Comparisons between different groups were carried out by one-way analysis of variance (ANOVA) followed by Duncan’s Multiple Range test for post hoc analysis using SPSS software version 15. The level of significance was set at P ≤ 0.05. Graph pad software Instat (version 2) was used for making graphs.

### Data Availability

All data generated or analysed during this study are included in this published article.

### Ethics approval

Ethics approval and consent to participate this study was approved by the Animal Use and Care Committee at Faculty of Veterinary Medicine, University of Sadat City, Egypt.

## Results

### Serum biochemical findings

Activities of serum ALT, AST, ALP and LDH were significantly elevated (p ≤ 0.05) in rats of Irradiated group that exposed to whole body irradiation in comparison with control. Serum total cholesterol, triglycerides, low LDL-C and VLDL-C concentrations were elevated (p ≤ 0.05) in irradiated animals, while HDL-C showed significant reduction. Pretreatment of animals of Group 4 with Date syrup caused considerable improvement of serum enzymes and lipid profile values (Table 1).

**Table 1:** Blood biochemical parameters in irradiated rats with or without administration of Date syrup.

Parameter	Control	Irradiated	Date syrup	Irradiated + Date syrup
ALT U/L	42.2 ± 2.396^c^	62.6 ± 2.502^a^	39.6 ± 2.839^c^	52.6 ± 3.311^b^
AST U/L	67.6 ± 1.691^c^	88 ± 1.673^a^	65 ± 1.140^c^	83 ± 1.924^b^
ALP U/L	67.2 ± 2.131^c^	99 ± 2.608^a^	64.6 ± 2.358^c^	77.8 ± 2.905^b^
LDH U/L	916.6 ± 24.116^c^	1097.6 ± 28.285^a^	905.6 ± 25.582^c^	1008 ± 29.11^b^
Cholesterol (mg/dl)	65.6 ± 3.906^bc^	79.2 ± 3.262^a^	62.8 ± 3.216^c^	75 ± 3.131^ab^
Triglycerides (mg/dl)	46.2 ± 2.989^c^	71.6 ± 2.786^a^	44.8 ± 2.354^c^	58.8 ± 2.518^b^
HDL-C (mg/dl)	42.8 ± 1.934^a^	28.6 ± 1.123^c^	46 ± 1.378^a^	36.8 ± 1.497^b^
LDL-C (mg/dl)	18.24 ± 1.849^c^	40.48 ± 3.226^a^	13.44 ± 3.154^c^	30.56 ± 3.341^b^
VLDL-C (mg/dl)	9.24 ± 0.598^c^	14.32 ± 0.557^a^	8.96 ± 0.471^c^	11.76 ± 0.504^b^

Values are presented as mean ± SE.

Means in the same row followed by different letter superscripts are significantly different at (P < 0.05).

### Oxidant/antioxidant biomarkers findings

**Figure 1:**
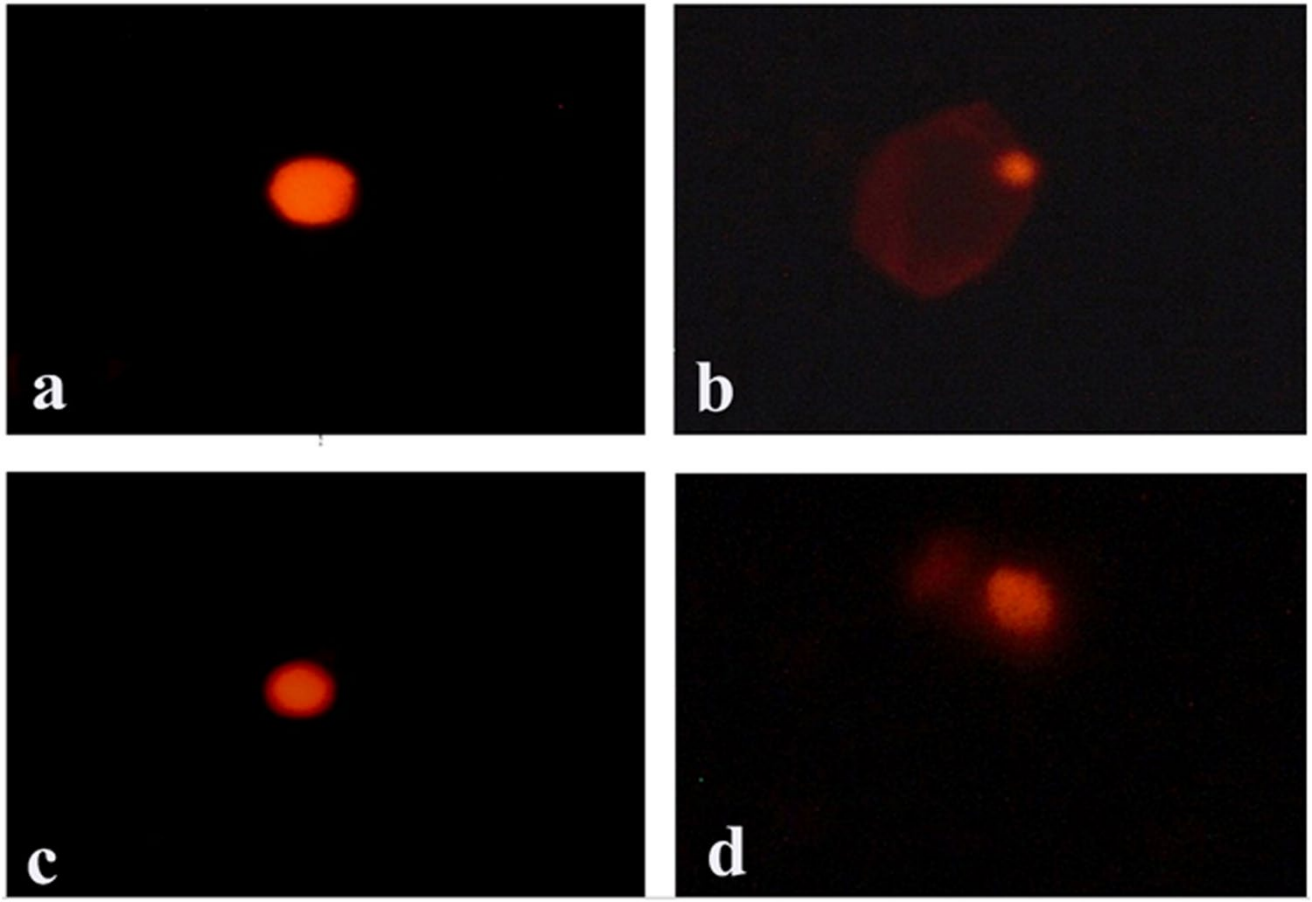
Comet assay in hepatic tissue of different groups. (**a**) Group 1 (control) showing circular intact DNA; (**b**) Group 2 (irradiated animals) showed comet shape and tailed DNA; (**c**) Group 3 (Date syrup) showing circular intact DNA similar to control group; (**d**) Group 4 (Irradiation + Date syrup) showing moderately intact circular DNA.

Hepatic MDA (Table 2) and DNA strand breakage **(**Fig. 1**)** confirmed by its crosslinking with protein (DPCs) (Table 2) significantly increased (p ≤ 0.05) in Irradiated group compared to control. Moreover, hepatic GSH concentration and CAT activity were significantly reduced (p ≤ 0.05) in comparison to control. All these parameters were relatively improved and shifted toward the normal values in rats of Group 4 received Date syrup before irradiation **(**Table 2**)**.

**Table 2:** Oxidant/antioxidant biomarkers in control and different groups.

Parameters	Control	Irradiated	Date syrup	Irradiated + Date syrup
MDA nmol/g	37.07 ± 0.482^c^	67.09 ± 0.789^a^	35.09 ± 0.592^c^	58.56 ± 0.566^b^
DNA damage				
A-Commet assay:				
1-Tail Length (µm)	3.5 ± 0.15^c^	6.4 ± 0.35^a^	3.2 ± 0.25^c^	4.5 ± 0.28^b^
2-DNA% in Tail	5.01 ± 0.5^c^	9.14 ± 0.36^a^	5.05 ± 0.4^c^	7.52 ± 0.4^b^
3-Tail Moment	0.18 ± 0.018^c^	0.58 ± 0.036 ^a^	0.16 ± 0.018^c^	0.33 ± 0.02^b^
B-DPCs	48 ± 0.6^b^	52 ± 1.2^a^	47.7 ± 0.3^b^	51 ± 0. 6^a^
GSH mg/g tissue	80.23 ± 1.795^a^	49.62 ± 1.316^c^	83.05 ± 2.710^a^	66.45 ± 1.673^b^
CAT U/g tissue	3.72 ± 0.014^a^	3.61 ± 0.015^b^	3.72 ± 0 .017^a^	3.66 ± 0.020^b^

Values are presented as mean ± SE.

Means in the same row followed by different letter superscripts are significantly different at (P < 0.05).

### MMP-9 finding

**Figure 2:**
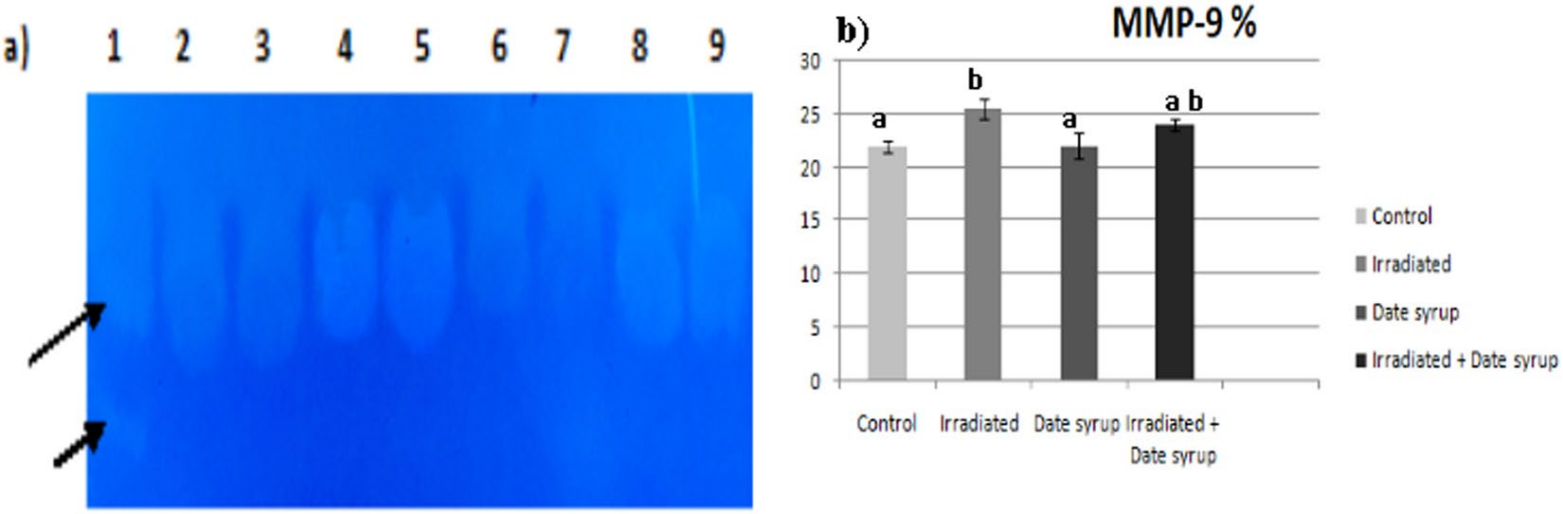
Activity of MMP-9 and gelatin zymography of enzyme activity in control and treated groups (**a**) A cropped zymogram gel for identification the activity of MMP-9. Lanes 2–3 = control; lanes 4–5 = Irradiated; lanes 6–7 = Date syrup; lanes 8–9 = Irradiated + Date syrup. Positive control shown in lane 1 is from baby hamster kidney cells transfected with active MMP-9 (86 kDa) and MMP-2 (66 KDa) that are indicated by arrows. (**b**) Quantification of enzyme activity shown as % of bands intensity, the zymogram was quantified by MyImage Analysis Software; Thermoscientific^TM^. Values are presented as mean ± SE. Means above column of histogram with different letter (a–b) are significantly different at (P < 0.05).

The activity of MMP-9 was significantly increased (P < 0.05) in Irradiated group compared to control group, while pretreatment with Date syrup before irradiation caused partial protection **(**Fig. 2**)**.

### Expression of TNF-α gene

**Figure 3:**
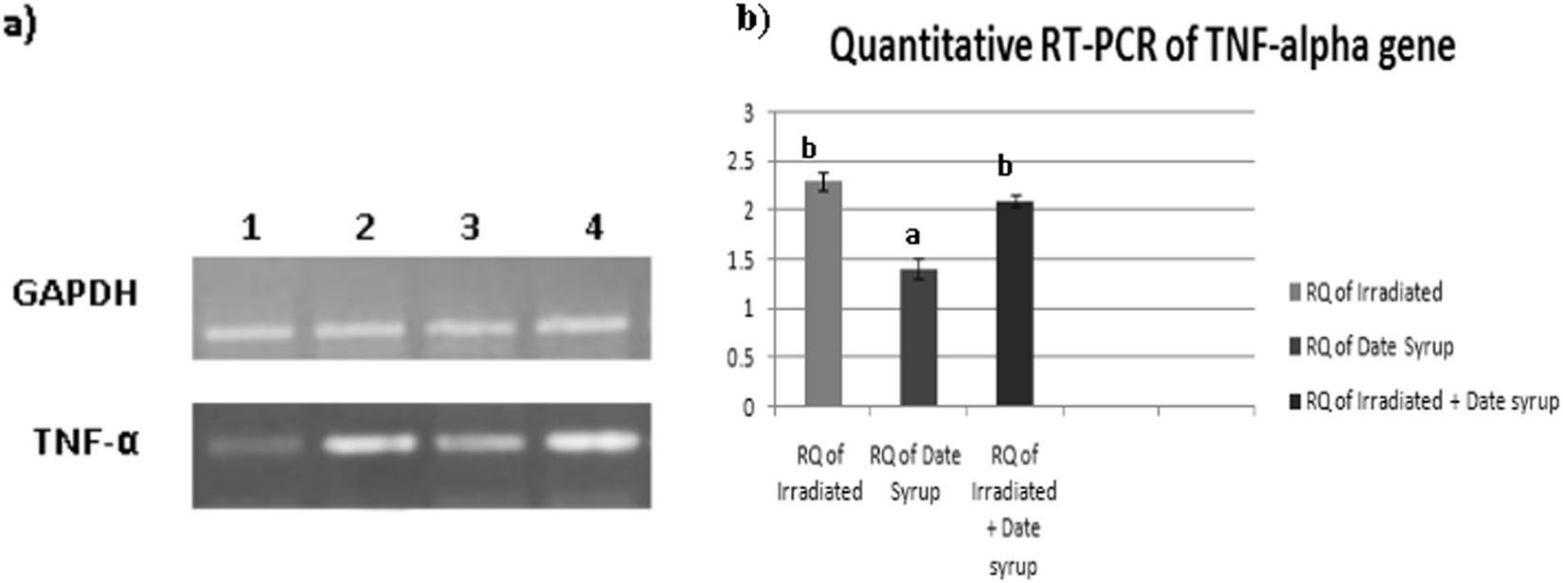
Relative quantitative expression (RQ) of TNF-α gene in liver of different groups. (**a**) Cropped gels identify the electrophoretic mobility of quantitative RT-PCR products of TNF-α and GAPDH (internal control) genes on two separate 2% agarose gels. Lane: 1= control; lane 2= Irradiated animal; lane 3= Date syrup animal; lane 4= Irradiated + Date syrup animal. (**b**) Evaluation of TNF-α gene expression in different groups. Real-Time PCR analysis was used to determine mRNA levels of TNF-α gene. The data were normalized to an endogenous reference, GAPDH and expressed as relative to control. Values are presented as mean ± SE. Means above column of histogram with different letter (a–b) are significantly different at (P < 0.05).

The relative expression of TNF-α gene significantly increased (P ≤ 0.05) in liver of Irradiated animals and animals pretreated with Date syrup before radiation to 2.3 and 2.1fold, respectively, in comparison to protected group, i.e. received Date syrup alone **(**Fig. 3**)**.

### Histopathological findings

**Figure 4:**
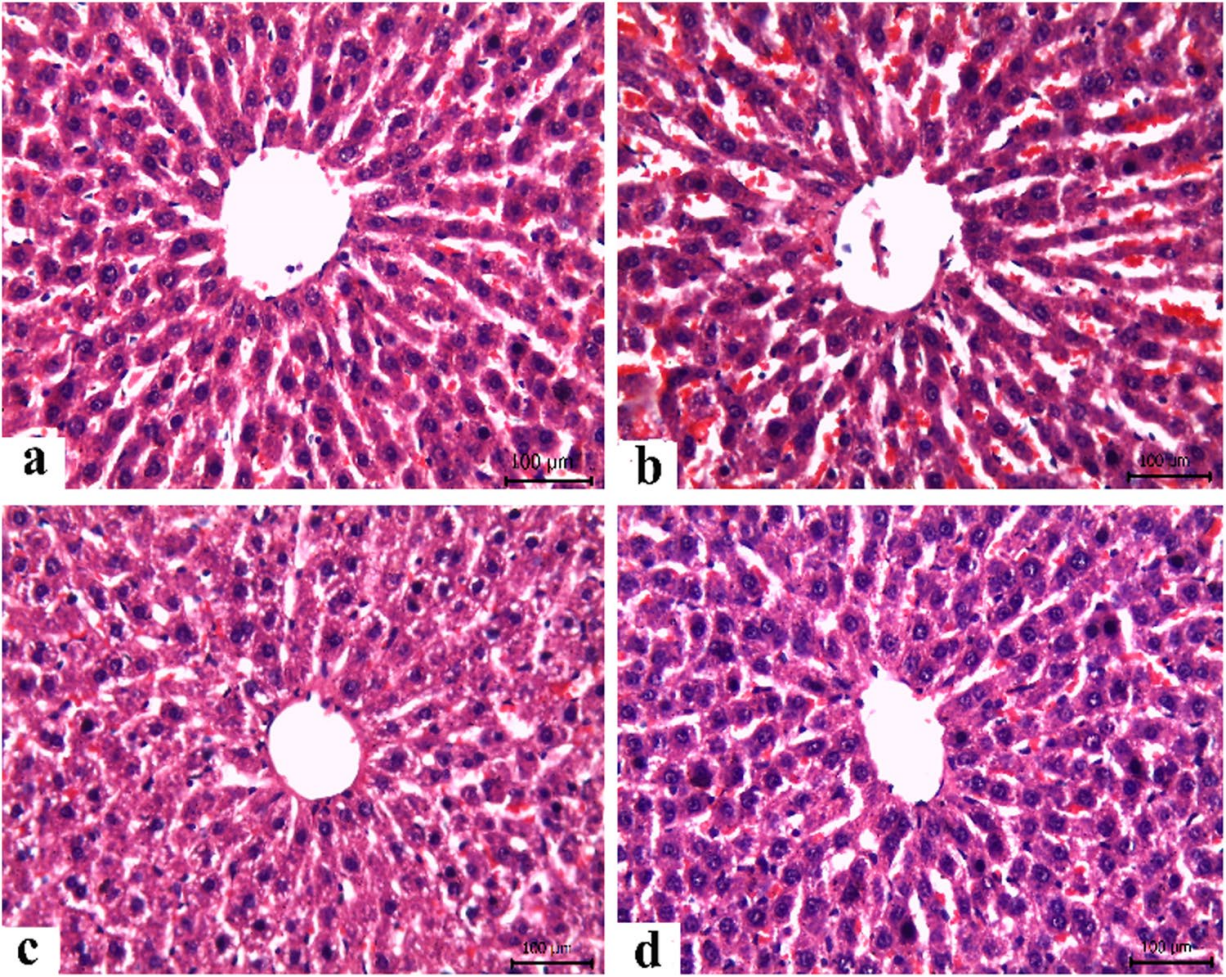
Histopathological findings. (**a**) Liver of control rat. (**b**) Liver of irradiated rat showing focal areas of degeneration, congested and dilated sinusoids. (**c**) Liver of a rat received dates syrup. (**d**) Liver of a rat pretreated with date syrup and exposed to radiation revealing slight congestion of sinusoids.

Table 3 presents the semi-quantitative scoring of histopathological lesions in liver of animals with different treatments. There were no histopathological alterations in the liver of control rats and the normal histological structure of the central vein and surrounding hepatocytes were observed **(**Fig. 4a**)**. Similarly, livers of rats received Date syrup showed no abnormalities **(**Fig. 4c**)**. In irradiated animals of Group 2 **(**Fig. 4b**)**, liver showed focal areas of degeneration, congested and dilated sinusoids. However, rats in that receiving irradiation followed Date syrup treatment revealed only slight congestion of sinusoids **(**Fig. 4d**)**.

**Table 3:** Semi-quantitative scoring of histopathological lesions in liver of all groups.

	Control	Irradiated	Date syrup	Irradiated + Date syrup
Degeneration	**−**	**++**	**−**	**−**
Congested and dilatated sinusoids	**−**	**+++**	**−**	**+**

−, None; +, slight < 20%; ++, moderate < 50%; +++, severe > 50% of examined sections.

## Discussion

Ionizing radiation produces its harmful effects through radiolysis which results in releasing of ROS in cells and depletion of cellular antioxidants including glutathione and enzymatic antioxidants. ROS can evoke the inflammatory response by increasing the expression of chemokines, cytokines and endothelial-leukocyte adhesion molecules^37^. The need for radioprotectives to protect normal tissues during radiotherapy motivated us to study the possible radioprotective effect of Date syrup (debis) in rats.

In the current investigation, gamma-irradiation of rats at 6 Gy increased the activities of serum ALT, AST, ALP and LDH. Similar findings were previously reported by Mansour^38^ and Salem *et al*.^39^. The elevation of serum transaminases is indicative of hepatocyte injury leading to increase in cell membrane permeability that facilitates the passage of cytoplasmic enzymes to blood. Hepatic ALP is present on the canalicular and luminal domain of the bile duct epithelium and levels rise because of increased synthesis and consequent release into the circulation due to biliary obstruction^40^. The increase in serum LDH can be attributed, like transaminases, to enzyme leakage through the damaged membrane of hepatocytes. In addition, it could be due to hypoxia resulting from hepatocyte injury^41^.

Our findings revealed that irradiation of rats induced significant increases in serum cholesterol, triglycerides, LDL-C and VLDL-C levels while HDL-C was reduced. This is consistent with earlier reports demonstrating hyperlipidemia as a consequence of whole body irradiation^21,42,43^. The irradiation-induced hyperlipidemia may be attributed to changes in liver lipid metabolism and serum lipoproteins and may be due to indirect effect of radiation through the release of different inflammatory mediators^43,44^.

Results of the present investigation demonstrated that whole body irradiation of rats at 6 GY significantly increased the level of liver MDA, while decreased hepatic glutathione reduced level and catalase activity. This was accompanied with histological alterations including focal areas of degeneration, congestion and dilated sinusoids. Similar findings were previously reported demonstrating that oxidative stress is induced by radiation^39,45,46^.

The increase in the level of MDA is a marker of increased rate of lipid peroxidation leading to tissue damage and consequent failure of the body natural antioxidants to detoxify the increased levels of free radicals. The reduction of liver concentration of glutathione, the major intracellular thiol of redox system, is indicative of consumption of this tripeptide in the detoxication of free radicals^47^. Similarly, the reduction of catalase activity in the liver is supposed to be due to increased utilization to detoxify H_2_O_2_ produced by lipid peroxidation. Catalase depletion may cause accumulation of H_2_O_2_ and superoxide radical triggering many harmful effects such as DNA and protein oxidation leading finally to cell death^48^.

Our results showed that exposure of rats to whole body radiation increased the DNA damage as indicated by changed comet parameters and increased percentage of DPCs. The DNA damage induced by exposure to radiation was previously demonstrated by comet assay^49,50^ and percentage of DPCs^51^. Alkaline comet assay is a sensitive technique to monitor DNA strand breaks and alkali labile DNA lesions, and is widely used to study genotoxicity and lesions in cellular DNA such as single and double strand breaks^52^.

DNA is usually associated with different structural and regulatory proteins in cells. Proteins are often covalently trapped on DNA; when cells are exposed to DNA-damaging agents pose the generation of DNA–protein crosslinks (DPCs)^53^. Our results demonstrated that whole body irradiation produced DNA damage in rats as evidenced by alterations of comet parameters and increase of the percentage of liver DNA-protein crosslinks. The formation of DPCs was originally reported in bacterial and mammalian cells that were heavily irradiated with ultraviolet light^54,55^. It was subsequently shown that DPCs are produced by a number of chemical and physical agents such as aldehydes^56^, metal ions^57^, anticancer drugs^58^ and ionizing radiation^59^. Moreover, DPCs need to be detected selectively in the background of many other DNA lesions, since DNA-damaging agents concurrently induce base damage, DNA strand breaks and DNA interstrand crosslinks along with DPCs^60^.

Our findings revealed elevated expression of TNF-α indicating that the radiation-induced tissue damage is associated with inflammatory processes^61^. Gaber *et al*.^62^ demonstrated increase in TNF-α expression in mouse brain exposed to radiation. Similarly, Lee *et al*.^61^ reported that irradiated rat brain showed marked up-regulation of mRNA and protein expression of the pro-inflammatory mediator TNF-α. This confirms the postulation that the destructive effect of radiation is mediated by proinflammatory cytokines production^63^.

Matrix metalloproteinases (MMPs) are involved in the contributory of tissues during embryonic development, cell migration, wound healing and tooth development^64^. However, deregulation of the balance between MMPs and TIMPs is responsible for diverse pathological conditions, such as rheumatoid and osteoarthritis, cancer progression, and acute and chronic cardiovascular diseases^11^.

MMPs can be activated by different stimuli in which proinflammatory cytokines (e.g., TNFα and IL-1β) play a role in the induction and propagation of inflammation and initiating an influx of neutrophils to the site of injury^9^. Likewise, several growth factors can initiate an intracellular signaling cascade leading to the activation of AP-1, NF_k_B, or ETS transcription factors, with consequent MMP transcription^10^.

In the present study, gelatin zymography demonstrated that MMP-9 activity significantly increased in irradiated group in comparison to control and protected groups. Administration of Date syrup ameliorated this enzymatic elevation by radiation. These results agree with the postulation that treatment with electromagnetic radiations can provoke ROS and reactive nitrogen species that trigger the activation of MMPs^65^.

These results agree with Patruno *et al*.^66^ who reported that electromagnetic field exposure to THP-1 cancer cells caused a weak increase in MMP-2 and -9 activities. Recent studies have implicated that radiation-induced MMP-9 led to enhanced tumor growth and metastasis^67^. Moreover, the effects of mechanical stimulations on MMP-9 gene expression and protein levels have been already demonstrated by the application of ultrasound on prostate cancer cells^68^, shear stress on breast cancer cells^69^ and pulsed electromagnetic field on chondrosarcoma cells^70^.

The present study demonstrated that Date syrup ameliorated the tissue damage induced by whole body irradiation of rats as evidenced by improvement of liver function and lipid profiles, and alleviation pro-inflammatory cascade. The antioxidant mechanisms of the body were enhanced as shown by elevated liver glutathione concentration and catalase activity with reduction of liver malondialdehyde level. In addition, Date syrup provided protection against the destructive effect of radiation on DNA as evidenced by improved comet assay parameters and reduced the percentage of DPCs. The histopathological observations were in congruence with the biochemical observations in liver and serum.

The hepatoprotective effect of Date fruit extract was previously reported in rats treated with dimethoate evidenced by decreased levels of the hepatic marker enzymes transaminases, alkaline phosphatase and lactate dehydrogenase^20^. This protective effect may be attributed to presence of many constituents in Date including selenium, anthocyanin, ferulic acid, caffeic acid, quercetin, chlorogenic acids, β-carotene, proanthocyanidins, apigenin and luteolin^13^.

The hypoglycemic and antihyperlipidemic activities of Date syrup recorded in the present study are consistent with previous studies of Ahmed *et al*.^71^ and Hasan and Mohieldein^14^. The antihyperlipidemic activity of Date may be attributed to presence of the phytochemical caffeic acid, β-sitosterol, proanthocyanidin, catechin, quercetin, anthocyanins and selenium^13^.

The protective effect of Date extract against the oxidative damage evoked by different toxicants has been previously reported in rats treated with carbon tetrachloride^17^, lambda cyhalothrin^72^ and dimethoate^73^. The Date phenolic compounds, anthocyanins, flavonoid glycosides and procyanidins are responsible for the observed antioxidant activity of Date fruit extract by detoxifying free radicals such as superoxide and hydroxyl radicals and thus inhibiting lipid peroxidation^74,75^. Presence of selenium mainly in the form of selenocysteine residues also contribute to the antioxidant effect of Date fruit as an integral constituent of ROS-detoxifying selenoenzymes; GPx and thioredoxin reductase^76^.

The observed anti-inflammatory effect of Date fruit as evidenced by reduction of hepatic TNF-α expression and serum MMP-9% is in the same line of Zhang *et al*.^77^, who reported that date fruit extract produces its anti-inflammatory activity through down regulation of cyclooxygenase-1 and cyclooxygenase-2 mediated by polyphenolic-rich compound, namely flavonoid glycoside. This is in accordance with previous studies reporting that many Date components have anti-inflammatory effect including flavonoids, β-carotene, proanthocyanidin, polyphenols and selenium^76,78^.

Our results demonstrated a radioprotective effect of Date syrup on the DNA of irradiated rats as shown by reduced DNA strand breakage and percentage of DPCs. This agree with the previous findings reporting that Date fruit extract exerted a dose-dependent inhibition of benzo(a)pyrene-induced mutagenicity on Salmonella with metabolic activation^79^. The radioprotective effect of Date syrup on the DNA may be attributed to presence of many constituents in Date fruit with antimutagenic properties including proanthocyanidins, anthocyanins, β-carotene, selenium and phenolic acids^80,81^.

## Conclusion

In conclusion, Date syrup can be effective in reducing radiation-induced hepatotoxicity, oxidative stress, inflammatory response and DNA damage. This makes the substance a potential supplement in the radiotherapy to protect normal cells from the destructive effects of radiation.

## Electronic supplementary material

Supplementary file
