# Supplementary material for: Radioprotective effect of Date syrup on radiation- induced damage in Rats
Source: Sci Rep. 2018 May 9;8:7423. doi: 10.1038/s41598-018-25586-3 (PMC5943437; doi:10.1038/s41598-018-25586-3)

Radioprotective effect of Date syrup on radiation- induced damage in Rats

Shimaa M. Abou-Zeid[1], Badr E. EL-bialy[1], Nermeen B. EL-borai[1], Huda O. AbuBakr[2]*, Abdel Monsef A. Elhadary[3]

1 Department of Forensic Medicine and Toxicology, Faculty of Veterinary Medicine, University of Sadat City. E-mail: shima10_a@yahoo.com, badr_elsaid10@yahoo.com, nermeenborai@yahoo.com

2 Department of Biochemistry and Chemistry of Nutrition, Faculty of Veterinary Medicine, Cairo University, Giza 12211, Egypt. E-mail: huda.omar@cu.edu.eg.

3 Atomic Energy Authority, Egypt.

*Correspondence author: Huda O. AbuBakr, Department of Biochemistry and Chemistry of Nutrition, Faculty of Veterinary Medicine, Cairo University, Giza 12211, Egypt. Telephone Number:02-01027799536 E-mail: huda.omar@cu.edu.eg

Fig 2: Gelatin zymography of enzyme activity in control and treated groups

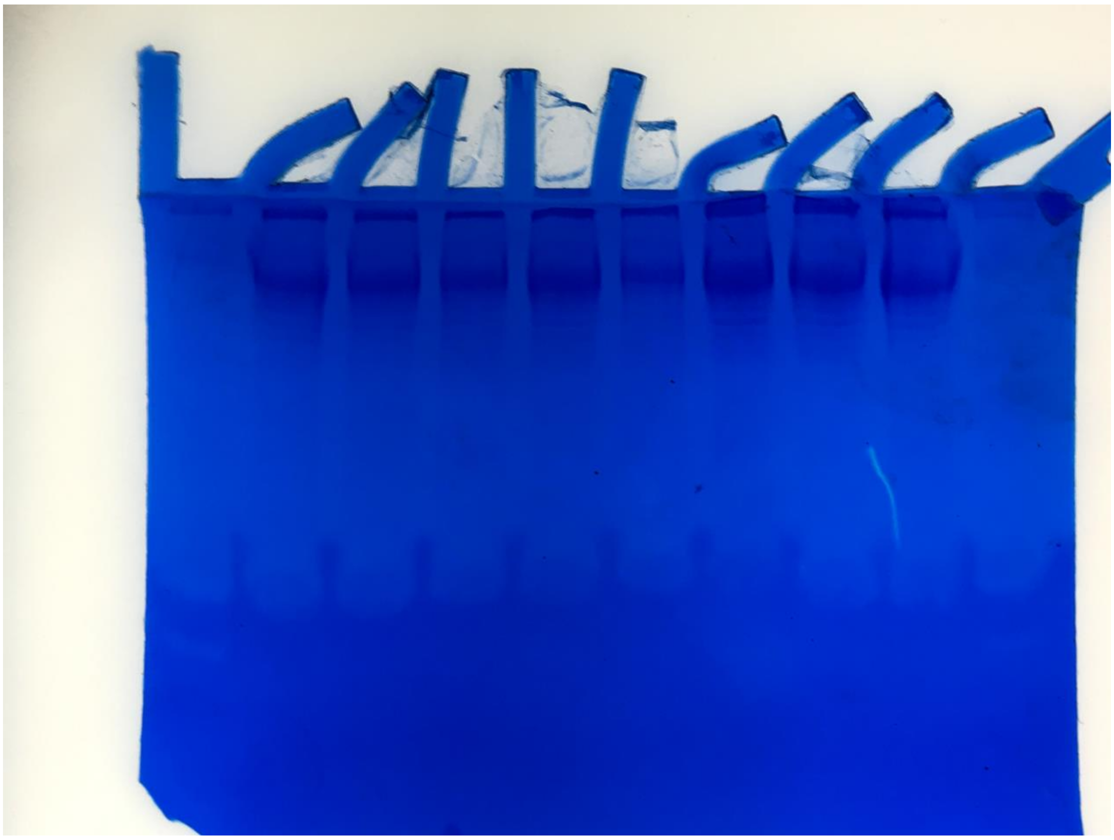

Supplement: Supplementary file 1 — Supplementary file [file 41598_2018_25586_MOESM1_ESM.pdf]
